# NAFLD and Extra-Hepatic Comorbidities: Current Evidence on a Multi-Organ Metabolic Syndrome

**DOI:** 10.3390/ijerph16183415

**Published:** 2019-09-14

**Authors:** Valerio Rosato, Mario Masarone, Marcello Dallio, Alessandro Federico, Andrea Aglitti, Marcello Persico

**Affiliations:** 1 Internal Medicine and Hepatology Division, Department of Medicine and Surgery, University of Salerno, 84081 Salerno, Italy; vrosato@unisa.it (V.R.); a.aglitti@studenti.unisa.it (A.A.); 2 Department of Precision Medicine, University of Campania “Luigi Vanvitelli”, 80131 Naples, Italy; marcello.dallio@gmail.com (M.D.); alessandro.federico@unicampania.it (A.F.); mpersico@unisa.it (M.P.)

**Keywords:** nonalcoholic fatty liver disease, metabolic syndrome, comorbidities, cardiovascular risk

## Abstract

Nonalcoholic fatty liver disease (NAFLD) is the most common cause of chronic liver disease worldwide and its incidence is definitely increasing. NAFLD is a metabolic disease with extensive multi-organ involvement, whose extra-hepatic manifestations include type 2 diabetes mellitus, cardiovascular disease, obstructive sleep apnea, chronic kidney disease, osteoporosis, and polycystic ovarian syndrome. Recently, further evidence has given attention to pathological correlations not strictly related to metabolic disease, also incorporating in this broad spectrum of systemic involvement hypothyroidism, psoriasis, male sexual dysfunction, periodontitis, and urolithiasis. The most common cause of mortality in NAFLD is represented by cardiovascular disease, followed by liver-related complications. Therefore, clinicians should learn to screen and initiate treatment for these extra-hepatic manifestations, in order to provide appropriate multidisciplinary assessments and rigorous surveillance. This review evaluates the current evidence regarding extra-hepatic associations of NAFLD, focusing on the pathogenic hypothesis and the clinical implications.

## 1. Introduction

Non–alcoholic fatty liver disease (NAFLD) represents a unique “challenge” for the hepatologist and is defined by the presence of hepatic steatosis in the absence of other causes for hepatic fat accumulation, including alcohol use, medication, or other causes of chronic liver disease [1]. As a result of the changes in dietary habits and the increased of sedentary lifestyle, its incidence has definitely increased worldwide over the last few years and, consequently, NAFLD can now be considered the most frequent liver alteration in the world [2]. NAFLD encompasses a wide pathological spectrum of liver injury, ranging from indolent steatosis to non-alcoholic steatohepatitis (NASH), where evolutionary liver fibrosis occurs resulting in cirrhosis along with other complications, including liver decompensation and hepatocellular carcinoma (HCC) [3]. Regarding the occurrence of HCC, it is worth note that NAFLD differs from other chronic liver diseases, in which the majority of patients develop HCC only after the onset of cirrhosis [4]. In fact, alarmingly, in NAFLD patients it has been reported that HCC occurs in about 50% of cases without the presence of such condition [5].

In the general population, a prevalence of NAFLD ranging between 17% and 33% was estimated, whereas, in obese and/or diabetic individuals, this prevalence reached 75% [2]. In fact, NAFLD is often referred to as the “hepatic manifestation” of metabolic syndrome (MetS) and, recently, growing evidence has highlighted the possibility that NAFLD may be a key driver in MetS; in fact, hepatic involvement could be just one component of a multi-organ syndrome that involves the cardiovascular, renal, and endocrine systems [6,7]. Since it has been widely demonstrated that NAFLD may precede the development of type 2 diabetes mellitus (T2DM) and MetS, the conventional theory of NAFLD as the “hepatic manifestation” of MetS has now been surpassed by evidence coming from more recent data indicating that NAFLD itself may possibly be the pathogenic determinant of MetS [7].

The liver-related mortality is increased about 5–10-fold in patients with NASH in realation to the degree of hepatic fibrosis, but the main causes of death are represented by cardiovascular disease and extra-hepatic malignancy, enphasizing the burden of metabolic systemic involvment of this disease [8,9]. Over the past decade, a wide range of extra-hepatic organ involvement has been demonstrated in NAFLD, and considerable evidence has recently defined a strong link between NAFLD and type 2 diabetes mellitus (T2DM), chronic kidney disease, atherosclerosis, and cardiovascular disease [10]. Furthermore, recent evidence has increased the broad spectrum of extra-hepatic involvement in NAFLD, including links with cardiomyopathy, cardiac arrhythmias, osteoporosis, obstructive sleep apnea, polycystic ovarian syndrome, male sexual dysfunction, psoriasis, hypothyroidism, urolithiasis, and periodontitis [10,11].

In this review, we evaluate the current evidence in literature regarding the pathogenic hypotheses at the basis of these connections and their clinical implications. A brief report of the key concepts analyzed in this review is summarized in Table A1.

## 2. Methodology

To evaluate the available evidence on NAFLD and extra-hepatic comorbidities, we performed a computer-based literature search on PubMed, Scopus, Google Scholar, and MEDLINE databases using the medical subject headings “NAFLD” and “extra-hepatic manifestation”. Then, the research further focused on the studies related to the extra-hepatic manifestations analyzed (i.e., T2DM, cardiovascular disease, chronic kidney disease, etc.). We limited the research to those published in English and, when possible, we excluded case reports or case series. Eligible studies were limited to those focusing on epidemiological, pathophysiological, clinical, and therapeutic aspects of extra-hepatic manifestations of NAFLD. 

## 3. Results

### 3.1. NAFLD and Type 2 Diabetes Mellitus/Insulin Resistance 

The pathophysiological relationship between NAFLD and insulin resistance (IR) is intricate and bidirectional; in fact most extra-hepatic manifestations that are correlated with NAFLD share IR as a common etiological factor [12]. A recent meta-analysis of 19 studies with 296,439 individuals estimated that NAFLD patients had a 2-fold higher risk of T2DM development with respect to the general population, and that this risk was further increased (4.7-fold) in the presence of advanced fibrosis [13]. In a study on 129 biopsy-proven NAFLD patients with a mean age of 51 years, the risk of T2DM development during a median follow-up time of 13.7 years was evaluated, showing that 78% of NAFLD subjects developed T2DM, with the risk 3-fold higher in NASH patients [14].

The exact mechanism of IR in NAFLD is still debated, but some key mechanisms have been described. They involve principally: (1) *Adiponectin*; (2) *pro-inflammatory cytokines* (*IL-6*, *TNF*-α); (3) *intracellular signaling intermediate* (*insulin receptor substrate* (*IRS*); (4) *protein kinases* (*Jun N-terminal kinase and inhibitor kappa beta kinase*); (5) *lipotoxicity* derived by *ceramides* and *diacylglycerols*; (6) *impaired gut microbiota composition*; (7) *mitochondrial dysfunction.*

*Adiponectin*, one of the most important adipocytokines, is involved in glucose and lipid metabolism, acting also as an anti-inflammatory and anti-atherogenic factor [15]. High concentrations of adiponectin were negatively associated with serum levels of low-density lipoprotein and triglycerides and positively with high-density cholesterol [16]. Therefore, the adiponectin concentration as inversely associated with IR and its levels were found to be lower in NAFLD patients [17]. Its activity protects against IR, enhancing fatty acid oxidation and glucose utilization and suppressing fatty acid synthesis [18]. *Pro-inflammatory cytokines*, e.g., *interleukin-6* and *TNF*-α, which are overexpressed in NAFLD, attenuate mediated signaling through the activation of various stress-related *protein kinases*, including *Jun N-terminal kinase (JNK)* and *inhibitor kappa beta kinase (IKK*) [19]. Similarly, the accumulation of *lipid metabolites*, e.g., *ceramides* and *diacylglycerols*, due to the increase in free fatty acid metabolism in NAFLD, activates numerous kinases, e.g., JNK and IKK, influencing insulin signaling via *IRS* [20]. The association between *gut microbiota* composition (usually impaired in NAFLD patients) and insulin sensitivity was evaluated, demonstrating an induction in IR [21,22]. Moreover, the improvement of IR after modulation of gut dysbiosis with antibiotic and probiotic treatments was also demonstrated, as was an increase in insulin sensitivity induced by gut microbiota transplantation from lean human donors to subjects with metabolic syndrome [23,24]. Finally, *mitochondrial dysfunction*, a common finding in NAFLD, probably due to excessive beta-oxidation of free fatty acids in the first stages of the disease, leads to reactive oxygen species (ROS) production as a result of successive blockage of fatty acid beta-oxidation, which is characteristic of later stages of NAFLD [25]. The resulting accumulation of *long-chain acylcarnitines*, *ceramides,* and *diacylglycerols*, derived by incomplete beta-oxidation, and *lipotoxic intermediates* may directly alter insulin signaling [26,27].

Established T2DM may promote NAFLD progression to cirrhosis and the development of HCC. Furthermore, the presence of T2DM defines an increase in all-causes and liver-related mortality in patients with NAFLD [12,27]. The liver is one of the main organs involved in the alteration of glycemic control during insulin resistance; in fact, patients with both NAFLD and T2DM were shown to have poor glycemic control compared to patients with only T2DM [28]. It was also noted that microvascular diabetic complications, such as retinopathy and nephropathy, were significantly more frequent in T2DM patients with concurrent NAFLD independent of confounding factors, including age, sex, body mass index (BMI), hypertension, smoking status, and medication use [29]. More interestingly, these same findings were showed in type 1 diabetic patients with ultrasound-diagnosed NAFLD, confirming that NAFLD is involved in the pathogenesis of renal and retinal complications, probably through the release of pathogenic pro-inflammatory mediators such as advanced glycated end-products, reactive oxygen species (ROS), TNF-α, and TGF-β [30]. Therefore, careful surveillance of macro- and microvascular complications should be provided to patients with coexisting T2DM and NAFLD. Moreover, in diabetic patients, the presence of NAFLD should be always investigated, bearing in mind that the majority of NAFLD diabetic patients do not present alteration in liver enzymes; thus, if there is suspicion for advanced liver disease based on non-invasive fibrosis scores (aminotransferase-to-platelet ratio index, APRI; fibrosis-4 index, FIB-4) or transient elastography (TE), a histological diagnosis should also be taken into account [31,32,33]. 

Currently, specific pharmacological treatments for NAFLD have not yet been approved, and most of the proposed interventions are the same as those commonly used for the treatment of T2DM, as proof of the high grade of interplay between these two conditions. 

Metformin, the first line of treatment in T2DM, has shown beneficial effects in patients with NAFLD, including a decrease in the amount of fat in the liver and an improvement in metabolic parameters and serum levels of aminotransferases [34]. In a recent study on a murine model, metformin treatment reduced markers of inflammation and lipoperoxidation in the liver and, at the same time, attenuated the loss of tight junction proteins in the small intestine, resulting in a decrease in the translocation of bacterial endotoxins in portal plasma and therefore in a protective effect upon NAFLD onset [35]. Furthermore, in a recent study on 42 NAFLD non-diabetic patients, significant reductions in hepatic steatosis and fibrosis were shown, which were evaluated by controlled attenuation parameter (CAP) and liver stiffness after five months of treatment with metformin and diet, compared to patients treated with diet alone [36]. Contrarily, a meta-analysis of 17 randomized controlled trials (RCTs) showed that 6–12 months of metformin plus lifestyle intervention did not improve liver histology or aminotransferases among NAFLD diabetic patients, compared with lifestyle intervention alone [37].

Thiazolidinediones (TZD), such as pioglitazone and rosiglitazone, modulate insulin sensitivity via activation of peroxisome proliferator-activated receptor (PPAR)-y. Several RCTs evaluated the efficacy of these drugs on the histological and clinical features of NAFLD, but the resulting evidence is controversial. Three meta-analyses agreed on the beneficial effects of TZD on lobular inflammation, but not on a clear improvement in liver steatosis or fibrosis [38,39,40]. Moreover, the long-term safety of TZD in non-diabetic NAFLD patients has not yet been established. 

Similarly, the glucagon-like peptide-1 analogue liraglutide was evaluated for treatment of NAFLD, showing a significant improvement in liver enzymes with good tolerability [41]. Furthermore, in a recent multi-center phase 2 RCT on NAFLD biopsy-proven patients, a significant improvement in steatosis and hepatocyte ballooning was shown after 48 weeks of liraglutide treatment (1.8 mg/day), but no significant differences in lobular inflammation and NAFLD fibrosis score were demonstrated [42].

### 3.2. NAFLD and Cardiovascular Disease

Cardiovascular disease (CVD) is the leading cause of death among patients with NAFLD, which commonly presents the classical CVD risk factors, including insulin resistance, hypertension, atherogenic dyslipidemia, and obesity [2]. Besides these shared risk factors that link CVD and NAFLD, evidence in NAFLD patients identified many non-traditional and emerging CVD risk factors, including pro-inflammatory cytokines (PCR, IL-6, TNF-α), procoagulant factors (fibrinogen, plasminogen, vascular adhesion molecules), and hyperuricemia [43,44,45]. In fact, it was observed that NAFLD was significantly associated to ischemic vascular events regardless of the presence of common metabolic risk factors, conferring an additional risk of premature CVD in patients with NAFLD alone [46,47].

Several studies reported that NAFLD was linked with an increase in *carotid intima–media thickness*, a validated tool for the assessment of atherosclerosis and risk of CVD events in asymptomatic patients, with a 3.7-fold higher likelihood of having carotid plaques in NAFLD patients compared to controls [48]. NAFLD was also associated, regardless of the presence of metabolic risk factors, with an increased value of *cardio-ankle vascular index*, a novel score of arterial stiffness that is associated with coronary heart disease and stroke [49]. Similarly, in a recent meta-analysis of 12 studies (with a total of 42,410 subjects), NAFLD was significantly related to a higher *coronary artery calcium (CAC) score,* a CT-based score for the quantification of coronary atherosclerosis, defining a higher risk of subclinical atherosclerosis [50]. Comparative studies between CAC scores and carotid intima–media thickness have not yet defined which is the most accurate predictor of CVD events, although recent evidence has shown a greater reliability of the CAC evaluation [51].

In a cohort study of 612 patients who underwent coronary angiography, fatty liver was associated with a significant increase in cardiovascular disease, with >50% of patients having stenosis in at least one coronary artery; this association remained significant after adjustment for metabolic factors and alanine aminotransferase levels [52]. In a longitudinal study among 1732 subjects without CAC evaluated with a baseline CT scan, NAFLD was correlated significantly with the initial development of coronary calcification, and the ultrasound severity of NAFLD was associated with CAC in a dose-dependent fashion [53].

*Gamma glutamyl transpeptidase (GGT)* levels, one of main markers of NAFLD, were associated with cardiovascular disease and stroke, even in subjects with low or moderate cardiovascular risk [54]. Indeed, GGT has been isolated from atherosclerotic plaques and it seemed to directly contribute to the progression of atherosclerotic lesions by inducing oxidative stress [55,56]. 

A recent meta-analysis of 16 studies with a total of 34,043 adult individuals showed a higher risk of CVD events during a median follow-up period of 6.9 years in NAFLD patients compared to controls, with a random-effect odds ratio(OR) of 1.64 overall, and of 2.58 in “more severe” NAFLD cases [57]. 

Moreover, in regard to CVD mortality, a study of 229 patients with biopsy-proven NAFLD, evaluated in a mean follow-up time of 26.4 years, showed an increased risk of death for CVD and liver-related disease compared to the reference population [9]. Finally, in a recent multi-national study of 458 patients, higher CVD mortality was reported in NAFLD patients with bridging fibrosis compared to those with cirrhosis, in whom the predominant cause of death was due to liver-related events [58].

Even among liver transplant (LT) recipients for NASH, cirrhosis was demonstrated to predispose patients to a higher risk of long-term CVD-specific mortality compared to non-NASH cirrhosis [59]. A higher risk of CVD events <1 year after LT was found in NASH cirrhosis compared to patients transplanted for alcohol-induced cirrhosis (26% versus 8%, with an odds ratio of 4.16) [60]. Similarly, even in comparison with all other etiology of cirrhosis, NASH patients showed the highest risk of CVD events, at 1 and 3 years after LT (15.3% and 19.3%, with a hazard ratio of 2.3), while primary sclerosing cholangitis (PSC) and primary biliary cholangitis (PBC) had a lower risk of CV events after three years (4.5%) [61].

Therefore, based on this strong evidence, NAFLD patients should be carefully monitored for CVD events by assessing historical CVD risk factors (family history of premature CVD (males <55; female <65), smoking, diabetes), evaluating yearly blood pressure when the patients are not already hypertensive, evaluating fasting lipid profiles, intervening in modifiable risk factors, and considering statins for primary prevention in patients aged 40–79 years with a 10-year risk >7.5%, according to the American Heart Association/America College of Cardiology prevention guidelines [62].

### 3.3. NAFLD and Cardiac Dysfunction (Cardiomyopathy and Cardiac Arrhythmias)

Besides the accumulating evidence regarding the adverse effects of NAFLD on coronary atherosclerosis, this multi-organ disease seems to affect all other anatomical structures of the heart, conferring an increased risk of *cardiomyopathy, cardiac valve calcification,* and *arrhythmia* [63].

Several small studies showed a significant correlation between NAFLD and *impaired diastolic function* or *abnormal left ventricular structure* and, in a recent cross-sectional study on 2713 middle-aged asymptomatic adults, NAFLD was strongly correlated to *subclinical myocardial remodeling and dysfunction*, regardless of the presence of traditional heart failure risk factors, including hypertension, dyslipidemia, diabetes, and obesity [64,65]. In a recent case-control study of 308 patients with NAFLD, hepatic fibrosis (assessed by transient elastography, TE) and steatosis (assessed by CAP) were independently associated with *larger left atrial volume*, *left ventricular diastolic dysfunction,* and *lower myocardial glucose intake*, evaluated with fluorodeoxyglucose - positron emission tomography (FDG-PET) [66]. Similarly, other studies of biopsy-proven NAFLD patients reported a graded relationship between *myocardial dysfunction* and the severity of the NAFLD histology, suggesting that, since myocardial alterations precede the onset of cirrhosis by a long time, myocardial changes may not be a direct consequence of the development of portal hypertension [67,68]. Subsequently, in a study on 1058 patients with heart failure who were followed up for a mean period of three years, the severity of NAFLD (assessed by the Fibrosis-4 score) was independently associated with *left ventricular diastolic dysfunction*, *larger atrial volume,* and *higher all-cause mortality* [69].

Some studies highlighted, independently of multiple cardio-metabolic risk factors, an association between NAFLD and *aortic valve sclerosis* and *mitral annular calcification*, conditions that correlated with poor cardiovascular outcomes and cardiac arrhythmias [70,71].

*Atrial fibrillation (AF)* is the most frequent sustained arrhythmia and some large-cohort studies have consistently documented that NAFLD is independently associated with an increased risk of incidence of atrial fibrillation [72,73]. In a cohort study of 702 patients with T2DM, an increased risk of atrial fibrillation (OR = 3.04) in NAFLD patients was reported, regardless of clinical risk factors for AF. In a subsequent prospective study conducted by the same investigators in a cohort of 400 T2DM patients followed for 10 years, NAFLD was associated with a four-fold increased risk of incidence of AF [74,75]. On the same issue, a recent meta-analysis of five cohort studies with a total of 238,129 participants showed a nearly two-fold increased risk in prevalence and incidence of atrial fibrillation [76].

All of this research suggests that patients with NAFLD have a significant risk of developing heart failure, calcifications of the aortic and mitral valves, and cardiac arrhythmias, particularly atrial fibrillation. However, further perspective and interventional studies are needed in order to define the pathophysiological correlations between NAFLD and these pathological conditions, and to establish the appropriate lines of intervention.

### 3.4. NAFLD and Chronic Kidney Disease

Chronic kidney disease (CKD) is defined as the gradual loss of kidney function, evaluated as the decrease in estimated glomerular filtration rate (eGFR) to less than 60 mL/min/1.73 m^2^, abnormal proteinuria, or overt proteinuria [29]. CKD is particularly frequent among NAFLD patients, ranging approximately from 20% to 50%, suggesting that NAFLD may accelerate the development and progression of CKD, regardless of common risk factors such as hypertension and T2DM [77,78].

In a meta-analysis of 23 studies with a total of 63,902 participants, the prevalence and incidence of CKD were evaluated in patients with simple fatty liver, NASH, and advanced fibrosis, showing a progressively increased risk of prevalence and incidence of CKD (2.1-, 2.5-, and 5.2-fold and 1.8-, 2.1-, and 3.3-fold, respectively), proving that the severity of NAFLD was directly associated with CKD [77].

A post-hoc analysis of an RCT of 261 patients with biopsy-proven NASH demonstrated an association between liver fibrosis and eGFR improvement after one year of lifestyle intervention, and also detected less improvement in renal function among patients without NASH resolution, suggesting the detrimental effect of NAFLD on kidney function [79]. Emerging pathophysiological mechanisms linking NAFLD and CKD included the dysregulation of *angiotensin-converting enzyme (ACE)-2*, *nutrient/energy sensor sirtuin-1, and AMP-activated kinase* and the impairment of anti-oxidant defense mediated by *nuclear factor erythroid 2-related factor (Nrf)-2* [80]. In obese patients, increased activity of the renin–angiotensin system was shown due to adipocyte production of ACE and angiotensin (ang)-2 [81]. Ang-2 has detrimental effects on both the liver (promoting insulin resistance, de novo lipogenesis, mitochondrial dysfunction, ROS production, and pro-inflammatory cytokine production) and kidneys (enhancing renal ectopic lipid deposition, oxidative stress, inflammation, and fibrosis) [82,83]. *ACE-2* acts as counter-regulator of this mechanism, degrading ang-2 in ang(1–7), which oppose biological activity to ang-2. ACE-2 expression is down-regulated in obesity and in experimental high-fat-induced NASH [84,85]. *Sirtuin (SIRT)-1* has up- and down-regulation gene transcription activity resulting in amelioration of glucose and lipid homeostasis in liver, muscle, and adipose tissues [86]. Furthermore, SIRT-1 activity has anti-oxidant and anti-inflammatory effects on liver and kidney tissues, which play crucial roles in NAFLD and CKD [87,88]. Nrf-2 is a transcription factor that up-regulates numerous antioxidant and detoxification enzymes; its dysfunction has detrimental effects on NAFLD and CKD progression [89,90].

Increased dietary fructose intake has been demonstrated to induce and promote all features of metabolic syndrome, including endothelial dysfunction, oxidative stress, hepatic steatosis, and kidney disease. Besides the alteration of satiety due to inhibition of leptin activity and stimulation of dopamine in the mesolimbic system, fructose induces higher visceral fat accumulation and insulin resistance compared to an equal intake of calories derived from glucose. In fact, in the hepatocytes, fructose metabolism promotes ATP depletion and a subsequent increase in AMP degradation to uric acid, resulting in triglyceride accumulation. Consequently, uric acid may itself induce oxidative stress in mitochondria, further promoting fat accumulation and IR. Moreover, it also may also alter nitric oxide generation in endothelial cells, both directly and as a consequence of IR, resulting in endothelial dysfunction with detrimental effects on the kidneys [91]. As a matter of fact, it was shown that increased dietary fructose or sugar-sweetened soda consumption was associated with worsening liver fibrosis and renal function [92,93]. Besides kidney injury driven by NAFLD, evidence suggested that CKD may mutually contribute to the development and progression of IR and NAFLD. In animal studies, metabolic dysregulation mediated by CKD resulted in enhanced lipolysis and lipid mobilization with fat redistribution from the adipose tissue to the liver mediated by zinc a2-glycoprotein, which up-regulates perlipin and lipoprotein lipase and down-regulates the VLDL receptor [80,94]. Furthermore, CKD, through the accumulation of uremic toxic metabolites (urea, indoxyl sulfate, and trimethyl-amine-N-oxide) induces intestinal dysbiosis and systemic inflammation, promoting NAFLD and insulin resistance [95,96]. The alterations in tight junctions, induced by *ammonia and ammonium hydroxide,* enhances the passage of lipopolysaccharides, contributing to systemic inflammation and promoting the growth of urea-metabolizing microbial strains [97]. In a study on a murine CKD model, oral activated charcoal, which absorbs urea and urea-derived ammonia, reduced plasma endotoxin levels and other systemic inflammation mediators, including IL-6, TNF-α, and malondialdehyde, restoring the epithelial tight junction proteins [97]. These findings encouraged the hypothesis of the interconnection between NAFLD and CKD, both in the development and progression of these two disorders. Therefore, improvement of NAFLD may also improve CKD and the management of CKD may ameliorate the clinical outcomes of NAFLD patients.

### 3.5. NAFLD and Obstructive Sleep Apnea

*Obstructive sleep apnea (OSA)* is a disorder of sleep breathing characterized by *chronic hypoxia (CIH)* during sleep, induced by repeated partial or complete collapse of the upper airway [98]. The prevalence of OSA is estimated at 4%–5% for men and 2% for women, but this rate increases to 30%–50% in obese individuals [99]. Several research efforts suggested that OSA and CIH are independent factors of liver injury induction [100]. CIH, leading to oxidative stress, lipid peroxidation, and systemic inflammation, may play a part in the dynamic imbalance between oxidant and antioxidant agents that promote the progression of NASH through liver fibrosis [24,101].

In a recent meta-analysis of nine studies (2272 participants), it was demonstrated that OSA was related to the development and progression of NAFLD in terms of liver enzymes and histological alterations. In NAFLD patients, OSA was significantly associated with ballooning and hepatic fibrosis compared to controls, demonstrating that OSA may predispose patients to the progression of liver steatosis [102]. Similarly, in another meta-analysis, OSA was associated with the presence of NAFLD, with a two-fold increased risk of developing NAFLD in patients with OSA, and a two-fold increased risk of developing progressive NASH in patients with NAFLD who also had OSA [103]. The mechanisms behind these relationships between NAFLD and OSA are still unknown, but attention has been focused on the formation of ROS and lipid peroxidation mediated by CIH, leading to worsening liver damage [104,105,106]. 

OSA was also associated with an increased risk of intestinal damage, demonstrated by findings of a negative correlation of CIH with *zonulin* levels and positive correlations with total cholesterol, aspartate aminotransferase (AST), GGT, and high sensitivity C-reactive protein (CRP) [107].

Continuous positive airway pressure (CPAP) noninvasive ventilation is the “gold standard” for treatment for OSA, eliminating upper airway collapse and improving sleep fragmentation. Several studies investigated the impact of CPAP on hepatic steatosis, but the results obtained were controversial. A recent meta-analysis of five studies in obese adult patients (192 subjects) showed a statistically significant decrease in both AST and alanine aminotransferase (ALT) levels in OSA patients who had undergone CPAP therapy for at least three months, but these results were not found in subjects with an average duration of CPAP treatment of less than three months [108]. In a Taiwanese nationwide population-based study, with a total of 5214 OSA patients enrolled from 2000 to 2008 with a 10 year follow-up period, a lower cumulative incidence of liver diseases, defined as NAFLD or cirrhosis, was shown in the CPAP treatment group [109]. Similar results were found in a pediatric cohort of OSA patients with biopsy-proven NAFLD. After CPAP treatment of about three months, patients showed a statistically significant decrease in ALT and markers of metabolic syndrome (leptin, CRP, and alpha-tocopherol/total lipid ratio) [110]. On the other hand, recent studies showed that CPAP treatment had positive effects on insulin resistance on non-diabetic patients; furthermore, a meta-analysis of six RCTs involving 699 subjects demonstrated that CPAP therapy reduced the level of total cholesterol and triglycerides [111,112,113]. Moreover, two recent meta-analyses demonstrated that CPAP treatment significantly decreased the serum levels of inflammatory markers, including CRP, tumor necrosis factor-a (TNF-α), and IL-8, in OSA patients [114,115].

Nevertheless, despite these results, other RCTs yielded conflicting results; in a cohort of adult OSA patients, no difference was found in levels of liver injury markers after two months of CPAP treatment. Furthermore, in a similar, more recent RCT, no differences were found in liver steatosis and fibrosis, which were evaluated by the FibroMax test [116,117]. However, the duration of CPAP treatment was less than two months in these trials, so the authors concluded that the lack of favorable results was probably due to the short treatment course. Finally, another recent meta-analysis confirmed these findings, showing no difference in improvement of liver steatosis and markers of liver injury after CPAP treatment [118].

In conclusion, due to its hypoxic burden, OSA may play an important role in the development of NAFLD and in the progression of liver fibrosis, although NAFLD is a very heterogeneous disease and CIH has yet to be confirmed as one of a variety of physiological changes. CPAP treatment, the gold standard therapy for OSA, may improve liver injury and slow the progression of disease, probably due to its positive influence on insulin resistance and oxidative stress, but the duration of treatment may need to be long (three months or more), and more RCTs with longer treatment durations are needed to elucidate the real effects of CPAP on NAFLD.

### 3.6. NAFLD and Osteoporosis

*Osteomalacia and osteoporosis* are frequent complications in patients with chronic liver disease, especially in the end-stages; therefore, these conditions have been categorized by the term “*hepatic osteodystrophy”* [119]. 

*Osteomalacia* is a rare condition characterized by poor bone mineralization, but frequently reported in patients with advanced primary biliary cholangitis, especially in countries with scarce solar irradiation [120]. *Osteoporosis* is a systemic skeletal disease, characterized by the reduction of bone mass and the deterioration of bone microarchitecture, a common presentation in patients with chronic liver disease [119]. An increased risk of poor bone mineralization is generally linked to aging, but recently NAFLD was shown to be connected to an increased risk of osteoporosis, as well as obesity. In an Asiatic retrospective study involving 7797 Chinese individuals, NAFLD was associated with a 2.5-fold increased risk of osteoporotic fractures, exclusively in male subjects [121]. As is well known, estrogen plays a protective role in the development of osteoporosis, indirectly inhibiting osteoclast activity through the down-regulation of pro-inflammatory cytokines (IL-1, IL-6, TNF-α, M-CSF) and the up-regulation of TGF-ß, an inhibitor of bone resorption [122]. Therefore, studies on the influence of NAFLD on the development of osteoporosis were mainly conducted on post-menopausal women. Among post-menopausal women with NAFLD, a significantly lower lumbar bone mineral density (BMD) was observed than in those without NAFLD [123]. Similarly, another study demonstrated lower BMD in the hips and the femoral necks of elderly, non-obese men and post-menopausal women [124]. These associations remain significant after adjusting for other risk factors (age, BMI, waist circumference). However, simple fatty liver does not seem to be associated with osteoporosis; in fact, lower bone mass values were commonly found in subjects with liver steatosis associated with elevated serum alanine aminotransferase and high-sensitivity C-reactive protein levels, which are suggestive of non-alcoholic steatohepatitis [125]. In a recent study of 231 asymptomatic subjects (160 post-menopausal women), a negative correlation between BMD and liver fibrosis, which was assessed by TE, was shown among NAFLD patients. Moreover, using multivariate linear regression analysis, liver fibrosis was independently correlated with low BMD among NAFLD patients [126].

Furthermore, NAFLD and obesity may also compromise bone mineral acquisition in pediatric patients, shining attention on other potential risk factors of osteoporosis not associated with aging. In an Italian case-control study performed on 44 obese children with NAFLD diagnoses, a significantly lower lumbar spine (LS) BMD Z-score was found compared to those without NAFLD. Among NAFLD-afflicted obese children, the presence of NASH and liver fibrosis were evaluated by liver biopsy, showing a significantly lower LS BMD Z-score in children with NASH compared to those with simple steatosis, therefore pointing to the role of low-grade inflammation in the process of bone mass loss [127].

Similar results were found in another study performed on 38 children with biopsy-proven NAFLD, in whom more severe bone demineralization was found in children with NASH than in those with simple fatty liver [128].

Undoubtedly, NAFLD patients commonly exhibited lifestyle habits at risk for low BMD (sedentary behavior and lower levels of physical activity) [129], but evidence identified several pathophysiological mechanisms and possible mediating molecules that may interplay directly in the increasing of bone loss.

The role of vitamin D insufficiency in bone homeostasis is well-known, and in NAFLD patients lower serum levels of vitamin D compared to healthy controls were described [130]. 

In an animal study, the antifibrotic effect of 1,25(OH)2D3, the active form of vitamin D, through the inhibition of hepatic stellate cells and, thus, the down-regulation of TGF-ß and platelet-derived growth factor expression was shown [131]. The critical role of vitamin D deficiency in the development of NAFLD was also shown by another study using a high-fat-diet (HFD) mouse model, in which the association of the HFD and vitamin D deficiency was crucial in the establishment of the gut alterations and the systemic inflammation underlying insulin resistance and hepatic steatosis [132].

Furthermore, a study of an obesity-related NASH rat model demonstrated that artificial sunlight, which induced elevation of the active form of vitamin D(3), or vitamin D(3) supplementation ameliorated NASH progression, reduced hepatocyte apoptosis, and reduced inflammation [133]. Similarly, alterations in the *GH/IGF-1 axis* was a frequent finding in NAFLD or NASH adults, in whom growth hormone (GH) deficiencies are common. In hepatic cells, GH directly decreases lipogenesis, thereby inducing lipolysis which determines insulin resistance, while IGF-1 activity stimulates glucose uptake by favoring insulin signaling [134]. In NASH patients, a lower level of IGF-1 compared with healthy controls was shown, and this discrepancy coincided with a higher histological severity of liver fibrosis [135]. Furthermore, in a diet-induced NAFLD mouse model, the GH and IGF-1 supplementation induced a significant improvement in liver steatosis and inflammation [136]. The activity of IGF-1 was also involved in bone tissue maturation and in skeleton rebuilding, so low levels of IGF-1 were associated with a higher risk of hip and vertebrae fractures [137]. The disruption in the GH/IGF-1 axis may be related to the low bone mass density in NAFLD patients, but more studies are needed to explore the long-term consequences of this relationship.

The chronic inflammatory process, which is related to NAFLD pathogenesis, seems to be involved in the decrease of BMD. Cellular lipid overload, resulting in cell lipotoxicity, triggers an inflammation cascade mediated by the hepatic stellate and dendritic cells, generating multiple pro-inflammatory, pro-coagulant and pro-fibrogenic molecules. This so-called “*sterile inflammation*” is involved in local injury and results in liver fibrosis and inflammatory osteoporosis [138]. Tumor necrosis factor-α (TNF-α) is increased in NAFLD patients and TNF-α inhibition, obtained by administration of pentoxifylline, was shown to reduce hepatocyte inflammation together with normalization of liver function [139]. TNF-α is a factor that inhibits osteoblastogenesis in parallel with osteoclasteogenesis induction and, under inflammatory conditions, induces bone loss, emphasizing the role of other pro-inflammatory molecules like IL-6 and macrophage colony-stimulating factors. This evidence may indicate that inflammation links NAFLD and osteoporosis, but further studies are needed to explain this complex pathophysiological process.

*Osteopontin (OPN)* regulates bone mineralization by reducing growth and aggregation of calcium crystals. OPN is overexpressed in obese and/or NAFLD patients, and several studies were conducted in order to demonstrate that this may be the direct link between obesity-induced inflammation and osteoporosis [140,141,142,143]. In fact, in high-fat-diet mouse models, OPN expression was significantly upregulated and antibody-mediated OPN exerted a protective effect on macrophage infiltration and inflammatory liver injury [143].

*Osteoprogeterin* has a central role in bone turnover, inhibiting osteoclast differentiation and activation and promoting osteoclast apoptosis. The levels of osteoprogeterin were found to be decreased in patients with abdominal obesity, IR, and NAFLD [144].

*Adiponectin,* which is inversely associated with the presence and severity of NAFLD, also exerts an anti-osteoporotic activity promoting osteoblast differentiations and inhibiting osteoprogesterin secretion [145].

Despite the broad current evidence, the exact pathogenetic link between NAFLD and osteoporosis has not yet been well-established. Further clinical studies are needed to define the appropriate therapeutic approach in this category of patients.

### 3.7. NAFLD and Psoriasis

Psoriasis is a chronic, inflammatory, immune-mediated skin disease. Initiation and maintenance of psoriasis plaques depends on a patient’s innate and adaptive immunity. Psoriasis is one of most common dermatological diseases in Western countries and its prevalence is estimated to be at 2%–3% worldwide [146]. The involvement of psoriasis is not usually limited to the skin, but includes other manifestations such arthropathy, uveitis, and inflammatory bowel disease. Among psoriatic patients, beyond 40 years of age there is a high prevalence of metabolic syndrome; this correlation is directly proportional to the severity of psoriasis, independent of the presence of obesity [147,148]. Therefore, metabolic syndrome is related to both NAFLD and psoriasis and, consequently, the prevalence of NAFLD is increased in patients with psoriasis [149,150]. In a large prospective population-based cohort study of 2292 participants, the prevalence of NAFLD was 46.2% among patients with psoriasis, and even higher when compared to the prevalence among patients without psoriasis (33.3%), even after adjustment for alcohol use, alanine aminotransferase levels, and metabolic syndrome components [151]. Two recent meta-analyses confirmed these data, estimating a two-fold greater risk of NAFLD in patients with psoriasis than non-psoriatic controls, and this risk appeared to be greater in patients with more severe psoriasis or with psoriatic arthritis [152,153]. Furthermore, in a retrospective cohort study on 29,957 children with psoriasis, a higher risk of obesity, metabolic syndrome, NAFLD, and elevated liver enzyme levels compared to non-psoriatic children was found [154]. However, there are currently no epidemiological studies on the prevalence and incidence of psoriasis among NAFLD patients.

The exact pathophysiological mechanism linking psoriasis to NAFLD is still unclear, but IR, which has a central role in NAFLD, is a common finding in patients with psoriasis [155]. IR is strongly influenced by the activity of pro-inflammatory adipocytokines, as TNF-α, IL-6, and leptin, inducing a state of persistent low-grade inflammation that underlies the physiopathology of both NAFLD and psoriasis [146]. TNF-α, which plays a crucial role in psoriasis by increasing keratinocyte proliferation and angiogenesis, was found to be increased in human and animal models of NAFLD, contributing to IR and uncontrolled lipolysis [156]. In patients with psoriasis and NAFLD, the use of etanercept (an anti-TNF-α proteic drug) seemed to prevent hepatic fibrosis, reduce transaminases levels, and improve IR (measured by the homeostasis model assessment (HOMA) index), supporting the hypothesis that these two diseases are pathophysiologically connected through glucose homeostasis alteration and cytokine dysregulation [157]. A subsequent study on psoriatic patients treated for 24 weeks with etanercept showed a partial resolution of imbalance between pro- and anti-inflammatory cytokines, with a reduction in serum levels of adipocytokines [158]. Finally, after only one week of treatment with etanercept, a favorable modulation of insulin sensitivity, high density lipoprotein (HDL), Apo A1, and Apo B:Apo A1 ratio was demonstrated [159]. On this topic, further anti-TNF agents, such as infliximab and adalimumab, which are already used for the treatment of psoriasis, were evaluated in NAFLD patients. These preliminary studies showed favorable effects on liver inflammation and fibrosis, giving encouraging, but not definitive, results [158,159,160,161,162].

Leptin, which regulates the appetite and body weight, is increased in obese patients, in whom leptin resistance was described. In both NAFLD and psoriasis, higher serum levels of leptin were found, which seemed to promote development of fatty liver and mediate skin proliferative and anti-apoptotic processes in T-cells, increasing secretion of pro-inflammatory cytokines by keratinocytes [163,164].

Several pieces of research showed that anti-hyperglycaemic agents, such as GLP-1 receptor agonist, DPP-4 inhibitors, and thiazolidinendiones, exert anti-psoriasis effects independent of weight loss and glycemic control by targeting keratinocyte proliferation and skin inflammation [165]. Moreover, some reports suggest that treatment with pioglitazone and ursodeoxycholic acid may improve both psoriatic skin lesions and NAFLD liver injury, but further studies are needed to clarify the usefulness of such treatments [166,167,168].

Methotrexate is one of the most commonly used systemic treatments for the moderate-to-severe psoriasis and its liver toxicity has been repeatedly well-documented, reaching rates of about 60% among treated patients [169]. It would be interesting to evaluate whether patients that developed NAFLD were the same patients treated with methotrexate, but unfortunately these data are not reported in the studies. Therefore, methotrexate treatment should be carefully monitored in patients with psoriasis due to worsening of pre-existing steatohepatitis [146].

### 3.8. NAFLD and Periodontitis

Periodontitis is a chronic disease of the teeth characterized by a deficiency in immunological response to bacterial dental plaque, resulting in progressive loss of attachment, tooth mobility, and finally, tooth loss [170]. This dental disease does not seem to be confined exclusively to the oral cavity, with various evidence in the literature showing an association with other systemic diseases, including metabolic syndrome [171]. Furthermore, several pieces of research showed that periodontal treatment may improve glycemic control and HbA1c in diabetic patients [172,173,174]. Consequently, several recent studies highlighted a link between periodontitis and the risk of development of NAFLD, with an incident rate ratio of 1.28, as well as the risk of progression of NAFLD to liver fibrosis [175,176]. A cross-sectional study on a Japanese oral health-check population, showed a two-fold higher risk of NAFLD-affliction at ultrasound in patients with periodontal disease [177]. A recent meta-analysis of 12 studies involving 53,384 patients confirmed this association, noting the relevance of periodontal pathogens, especially *Porphyromonas gingivalis,* on NAFLD development and progression [178]. In a recent translational study, a positive correlation between fibrosis progression and antibody titers against *P. gingivalis* was shown in biopsy-proven NAFLD patients, as well as more severe steatosis and fibrosis in high-fat diet (HFD) mice with *P. gingivalis* odontogenic infection, compared to those without [179]. Similarly, another translational study showed a positive correlation of anti-*Aggregatibacter actinomycetemcomitans* antibody presence with visceral fat and HOMA-IR in 52 NAFLD patients, suggesting that alterations in the gut microbiota induced by *A. actinomycetemcomitans* may affect insulin resistance. In the same study, the high-fat diet (HFD)-fed mice infected with *A. actinomycetemcomitan* compared to controls (HFD mice without *A. actinomycetemcomitan* infection) showed impaired glucose tolerance, insulin resistance, and greater hepatic steatosis together with up-regulation of several genes, leading to increased glucagon and adipocytokine signaling upon liver microarray analysis [180]. Until now, the exact mechanism linking periodontitis and NAFLD is still unclear, but possible explanations include the association between the low-grade inflammatory response and the lipopolysaccharides and pro-inflammatory cytokines driven by periodontal pathogens, or the alteration in gut microbiota resulting from swallowed periodontal pathogens, such as *P. gingivalis* or *A. actinomycetemcomitan*, producing systemic inflammation involved in the development and progression of NAFLD [181,182]. Based on this hypothesis, periodontal treatment, which reduces the bacterial load, may ameliorate the course of this disease [183]. In fact, in a study of 150 biopsy-proven NAFLD subjects, a higher frequency of *P. gingivalis* infection compared to controls was demonstrated, and an improvement in liver function, evaluated using serum levels of AST and ALT, was obtained after three months of non-surgical periodontal treatment [183]. Nevertheless, this result was derived from the observation of only ten patients, therefore, no firm conclusions can be drawn. Furthermore, this study did not report whether restricted sugar/fructose intake was performed during the treatment period, which could have a better effect on NAFLD than any other therapy.

### 3.9. NAFLD and Hypothyroidism

The alterations in the concentration of thyroid hormones, which are generally involved in the regulation of energy expenditure, body fat distribution, lipid utilization, and glucose homeostasis are other potential factors that may contribute to the development of NAFLD [184]. In a cross-sectional study of 2324 patients, hypothyroidism was shown to be related to NAFLD, independent of relevant known metabolic risk factors and in a dose-dependent manner. This was the case even in the setting of subclinical hypothyroidism, where thyroid stimulating hormone (TSH) levels were in the upper range of normality [185]. In a recent study of 425 subjects with biopsy-proven NAFLD, the prevalence of NASH was significantly higher in patients with low thyroid function compared to those with normal TSH levels (52.4% vs. 37.2%). Using multivariate analysis, low thyroid function was independently related to the presence of NASH and advanced fibrosis, with a risk that was directly proportional to the increase in TSH levels [186].

A meta-analysis of 11 articles including 12,924 participants assessed the relationship between NAFLD and thyroid dysfunction, reporting a prevalence of hypothyroidism ranging from 15.2% to 36.3% among NAFLD patients [187]. Until now, little evidence has been provided regarding the pathophysiological mechanism that relates the decreased thyroid activity to NAFLD development and progression. Increased serum TSH levels induce liver triglyceride accumulation, mainly via up-regulation of SREBP-1 stimulated by TSH receptors, resulting in induction of NAFLD [188]. In a recent study of a methionine–choline-deficient murine model, thyroid hormone receptor knock-out mice showed an enhanced response to TGF-ß in hepatic stellate cells, suggesting a potential role of thyroid hormone signaling in hepatic fibrogenesis [189]. Other studies also demonstrated that low thyroid hormones levels may alter hepatic fatty acid composition and glycogen accumulation, decreasing the activity of acetyl-CoA carboxylase 1 and fatty acid synthase [190,191].

Furthermore, in two randomized controlled trials, improvement in serum liver enzymes and steatosis (evaluated by ultrasonography or by proton magnetic resonance spectroscopy) was shown after levothyroxine replacement treatment in NAFLD patients with subclinical hypothyroidism or euthyroidism [192,193]. In another recent RCT in adult patients with biopsy-proven NAFLD, a reduction in liver fat content (assessed by magnetic resonance imaging proton density fat fraction (PDFF)) after 12 weeks of treatment with MGL-3196 (a selective TSH receptor ß agonist) was demonstrated [194]. This additional evidence further supports the link between NAFLD and alterations in thyroid function.

Besides these data, a recent Spanish retrospective population study of 10,116 individuals found hypothyroidism in 9.1% of NAFLD patients with no significant differences with respect to controls, according to the presence of NAFLD [195].

Therefore, the hypotheses regarding the correlation between hypothyroidism and NAFLD are still speculative; further studies are necessary to elucidate the role of thyroid dysfunction on the progression of this multi-organ syndrome and the possible therapeutic implications.

### 3.10. NAFLD and Male Sexual Dysfunction

Erectile dysfunction (ED), defined as the inability to attain and/or maintain an erection satisfactorily during sexual acts, affects about 50% of men in the United States and approximately 322 million men worldwide [196]. The pathological alterations underlying ED have many of the same risk factors as MetS, including hypertension, dyslipidemia, physical inactivity, and visceral obesity [197]. NAFLD is recognized as the hepatic manifestation of MetS, therefore several studies evaluated its possible correlation with ED, identifying NAFLD as a risk factor for the presence of ED [198]. In a prospective study of 192 patients with NAFLD, ED was diagnosed in about 45% of patients; likewise, IR and low serum testosterone levels were identified as predictors of ED using multivariate analysis [199]. Unfortunately, the exact pathogenic link between these two diseases has not yet been established. In a study of an HFD-induced rabbit model of MetS, several genes related to inflammation, including TNF-α and IL-6, were negatively associated with maximal expression of acetylcholine-induced relaxation in the penis after development of NASH. Furthermore, a 15-fold increase in circulating levels of TNF-α was found, which was, using a multivariate model, the only significant association with penile acetylcholine responsiveness. Finally, the penile hypo-responsiveness to acetylcholine was partially normalized with testosterone and obeticholic acid (the selective farnesoid-X receptor agonist) treatment, and completely restored with infliximab (anti-TNF-α mAb) treatment [200]. Although this evidence is present in the literature, further studies are needed to clarify the association between NAFLD and ED, the subsequent clinical implications, and potential therapies.

### 3.11. NAFLD and Polycystic Ovarian Syndrome

Polycystic ovarian syndrome *(PCOS)* occurs in 5%–18% of women, and is characterized by hyperandrogenism symptoms, such as acne, hirsutism, oligomenorrhea, and amenorrhea [201]. In a small sample size study of 14 reproductive-aged female patients with biopsy-proven NAFLD, PCOS was diagnosed in 10 (71%), with 5 patients having documented fibrosis [202]. Several studies highlighted a higher risk of NAFLD in women with PCOS, and a recent meta-analysis of 17 studies confirmed this association, with 3.3-fold higher estimated risk. However, there was no association between PCOS and NAFLD in patients without hyperandrogenism [203]. In fact, PCOS is associated with insulin resistance and T2DM, and approximately 50% to 70% of women with PCOS exhibit IR which, in turn, may contribute to the hyperandrogenism that is responsible for the signs and symptoms of PCOS [204]. Confirming these findings, a Chinese study demonstrated that PCOS women with NAFLD had higher BMIs, insulin resistance levels, and triglycerides [177].

Several studies have shown that the presence of PCOS and hepatic steatosis is associated with insulin resistance and MetS [205], but a cross-sectional study of 54 Indian women found that IR levels were higher among women with PCOS and NAFLD compared to those with PCOS but without steatosis, proving the importance of IR in the pathophysiological mechanism involved in the development of NAFLD [206]. In a study of 14 young PCOS women with NAFLD, insulin sensitivity was measured using a euglicemic hyperinsulinemic clamp; an inverse association between serum ALT levels and insulin sensitivity was demonstrated, stressing the hypothesis that, in PCOS, the development of NAFLD is associated with IR [207]. Approximately 60% of PCOS women are considered obese, but this association with NAFLD appears to be independent of obesity. In a recent case-control study of 275 non-obese women with PCOS, NAFLD was significantly more frequent compared to controls without PCOS, and the level of androgenicity, which was assessed by free testosterone using the free androgen index, was associated with NAFLD, irrespective of age, lipid profile, and insulin resistance. This suggests an independent contribution of hyperandrogenism to the development of NAFLD [208]. 

From the analyzed data, it seems clear that these two pathologies share common risk factors, but many points still need to be clarified regarding their pathophysiological relationships. However, among women with PCOS, the presence of NAFLD should be investigated, even if optimal screening methods are not currently defined in this population. 

### 3.12. NAFLD and Urolithiasis

Urolithiasis is one of the most common urological diseases, characterized by the deposition of crystals in the renal medulla and in the genitourinary tract. Its prevalence has increased over the last few years, reaching 8% in the United States in 2010 [209]. Several metabolic disorders, including obesity, T2DM, and hypertension, are associated with the development of urolithiasis, thus suggesting that urolithiasis may be considered a metabolic disease [210]. Some recent studies identified NAFLD as a risk factor for the development of urolithiasis, estimating a higher prevalence of urinary calculi among NAFLD patients compared with healthy controls [211]. A recent systematic meta-analysis of 8 studies with a total of 238,400 participants estimated a significantly higher risk, about 1.8-fold, of urolithiasis among NAFLD patients [212]. The mechanism underlying this increase in risk is not completely defined, but possible explanations may be found in the common metabolic risk factors for both diseases and the role of systemic inflammation and oxidative stress. Hyperuricemia is a frequent finding among NAFLD patients, and it is well-known that insulin resistance may decrease urinary ammonia synthesis and excretion, resulting in lower urinary pH and creating favorable conditions for the formation of uric acid stones [213,214]. Furthermore, idiopathic calcium urolithiasis seems to be induced by altered oxidative stress, increasing renal non-albumin protein excretion and reducing urinary ammonia, resulting in higher hydroxyapatite crystallization and stone formation [215]. As touched on previously, NAFLD and MetS are characterized by persistent, low-grade inflammation and the resulting oxidative stress may damage the kidney parenchyma through lipid peroxidation of long-chain poly-unsaturated fatty acids, increasing the risk of calcium oxalate stone formation [216]. Despite this evidence, further studies are needed to clarify the connection between these pathologies; physicians should take into account the increased risk of urolithiasis in patients with NAFLD.

## 4. Conclusions

The clinical burden of NAFLD is not limited to liver-related morbidity and mortality, but involves multiple organs in a system fashion. The pathogenic link of NAFLD not only extends to common metabolic diseases, including T2DM, atherosclerosis, and cardiovascular disease, but also to diseases that are often not intuitively related to the metabolic imbalances found in such patients. In light of current evidence, greater awareness of these correlations is needed; therefore, extensive multidisciplinary screening should be provided to NAFLD patients, including CVD, CKD, OSA, hypothyroidism, osteoporosis, PCOS, urolithiasis, and periodontitis screening. The main therapeutic approaches for NAFLD patients should concern lifestyle modification (physical activity, weight loss, smoking cessation) and an early approach to pharmaceutical treatments (insulin sensitizers, lipid-lower agents) in order to impact extra-hepatic manifestations. In most of the studies analyzed, the extra-hepatic involvement of NAFLD was observed during a short follow-up period, therefore, the evaluation of these manifestations should be pursued as soon as possible.

## Appendix A

**Table A1:** Key concepts of extra-hepatic manifestations of nonalcoholic fatty liver disease (NAFLD).

Established Linkages	
Type 2 diabetes	IR is the common pathogenic mechanism for both T2DM and NAFLD and the severity of NAFLD is directly proportional to incidence of T2DM [16,17,18,19,20,21,22,23,24,25,26,27]. The mortality of T2DM patients is increased 2-fold in the presence of NAFLD [13,28]. The risk of macro- and microvascular diabetic complications is increased by presence of NAFLD [29,30].
Cardiovascular Disease Atherosclerosis Heart failure Dysrhythmia	The primary cause of mortality in NAFLD is cardiovascular disease [2,44,45,46,47,48]. A higher risk of cardiovascular events is related to more “severe” forms of NAFLD [58,59,60,61,62]. Furthermore, in NAFLD patients, the frequency of aortic valve sclerosis and atrial fibrillation is higher [71,72,73,74,75,76,77].
Chronic Kidney Disease	The risk of renal impairment is increased by the presence of NAFLD [78,79]. Improvement in hepatic disease also improves renal function [90].
Obstructive Sleep Apnea	OSA is significantly associated with more “severe” forms of NAFLD and the treatment of OSA with CPAP therapy seems to improve the liver injury [103,104,110,111,112,113,114,115,116,117,118,119].
Endocrinopathies Polycystic ovarian syndrome Hypothyroidism	Obesity and IR are common risk factors for both PCOS and NAFLD [207,208,209,210]. The prevalence of NAFLD is higher in patients with hypothyroidism; furthermore, low thyroid function seems to worsen the progression of liver damage, while replacement therapy may improve liver function [187,188,189,194,195,196,197].
Emerging Linkages	
Osteoporosis	NAFLD is associated with a 2.5-fold risk of osteoporotic fractures [122,124,125].
Urolithiasis	NAFLD patients have an almost doubled risk of developing urolithiasis, both for urate and calcium stones [213,214].
Periodontitis	Periodontitis is significantly associated with the presence of NAFLD and common periodontal pathogens seem to be able to influence the development and alterations of NAFLD [179,180,181,182,183,184].
Psoriasis	IR is a common risk factor for both psoriasis and NAFLD. Patients with psoriasis have a 2-fold increased risk of suffering from NALFD [151,152,153,154,155,156].
Male sexual dysfunction	NAFLD is an emerging risk factor for the development of erectile dysfunction [200,201].

CPAP: continuous positive airway pressure; IR: Insulin resistance; NAFLD: Nonalcoholic fatty liver disease; OSA: Obstructive sleep apnea; PCOS: Polycystic ovarian syndrome; T2DM: Type 2 diabetes mellitus.
